# Supplementary material for: Structure and function of the divalent anion/Na+ symporter from Vibrio cholerae and a humanized variant
Source: Nat Commun. 2017 Apr 24;8:15009. doi: 10.1038/ncomms15009 (PMC5413979; doi:10.1038/ncomms15009)
Supplement: Supplementary Information — Supplementary Figures, Supplementary Tables. [file ncomms15009-s1.pdf]

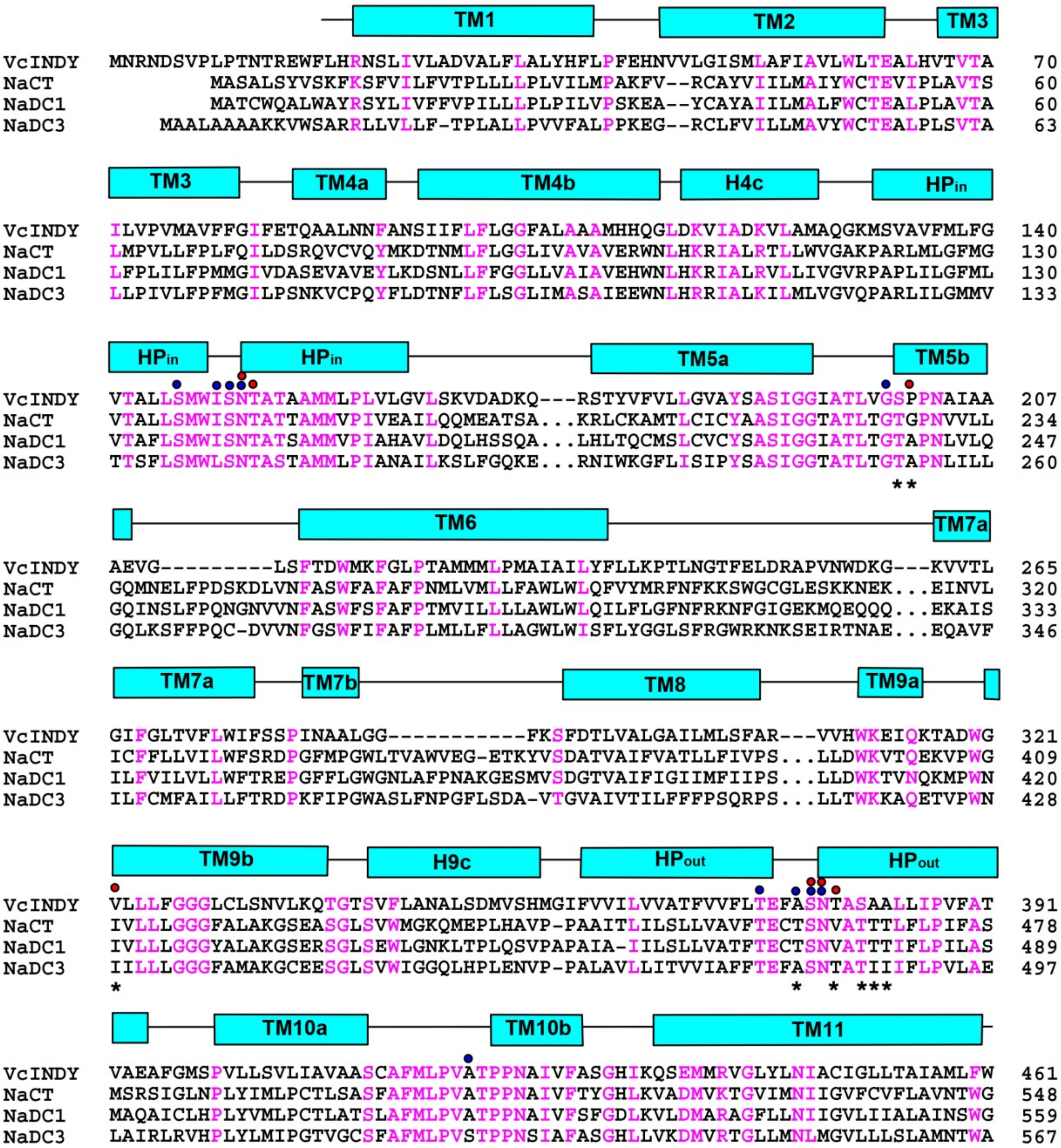
TM1
TM2
TM3
VcINDY MNRNDSVPLPTNTREWFLHRNSLIVLADVALFLALYHFLPFEHNVVLGISMLAFIAVLWLTEALHVTVTA 70
NaCT MASALSYVSKFKSFVILFVTPLLLLPLVILMPAKFV--RCAYVIILMAIYWCTEVIPLAVTS 60
NaDC1 MATCWQALWAYRSYLIVFFVPILLLPLPILVPSKEA--YCAYAIILMALFWCTEALPLAVTA 60
NaDC3 MAALAAAAKKVWSARRLLVLLF-TPLALLPVVFALPPKEG--RCLFVILLMAVYWCTEALPLSVTA 63
TM3
TM4a
TM4b
H4c
HPin
VcINDY ILVPVMAVFFGIFETQAALNNFANSIIFLFLGGFALAAAMHHQGLDKVIADKVLAMAQGKMSVAVFMLFG 140
NaCT LMPVLLFPLFQILDSRQVCVQYMKDTNMLFLGGLIVAVAVERWNLHKRIALRTLLWVGAKPARLMLGFMG 130
NaDC1 LFPLILFPMMGIVDASEVAVEYLKDSNLLFFGGLLVAIAVEHWNLHKRIALRVLLIVGVRPAPLILGFML 130
NaDC3 LLPIVLFPFMGILPSNKVCPQYFLDTNFLFLSGLIMASAIEEWNLHRRIALKILMLVGVQPARLILGMMV 133
HPin
HPin
TM5a
TM5b
VcINDY VTALLSMWISNTATAAMMLPLVLGVLSKVDADKQ---RSTYVFVLLGVAYSASIGGIATLVGSPPNAIAA 207
NaCT VTALLSMWISNTATTAMMVPIVEAILQQMEATSA...KRLCKAMTLCICYAASIGGTATLTGTGPNVVLL 234
NaDC1 VTAFLSMWISNTATSAMMVPIAHAVLDQLHSSQA...LHLTQCMSLCVCYSASIGGIATLTGTAPNLVLQ 247
NaDC3 TTSFLSMWLSNTASTAMMLPIANAILKSLFGQKE...RNIWKGFLISIPYSASIGGTATLTGTAPNLILL 260
**
TM6
TM7a
VcINDY AEVG---------LSFTDWMKFGLPTAMMMLPMAIAILYFLLKPTLNGTFELDRAPVNWDKG---KVVTL 265
NaCT GQMNELFPDSKDLVNFASWFAFAFPNMLVMLLFAWLWLQFVYMRFNFKKSWGCGLESKKNEK...EINVL 320
NaDC1 GQINSLFPQNGNVVNFASWFSFAFPTMVILLLLAWLWLQILFLGFNFRKNFGIGEKMQEQQQ...EKAIS 333
NaDC3 GQLKSFFPQC-DVVNFGSWFIFAFPLMLLFLLAGWLWISFLYGGLSFRGWRKNKSEIRTNAE...EQAVF 346
TM7a
TM7b
TM8
TM9a
VcINDY GIFGLTVFLWIFSSPINAALGG-----------FKSFDTLVALGAILMLSFAR---VVHWKEIQKTADWG 321
NaCT ICFFLLVILWFSRDPGFMPGWLTVAWVEG-ETKYVSDATVAIFVATLLFIVPS...LLDWKVTQEKVPWG 409
NaDC1 ILFVILVLLWFTREPGFFLGWGNLAFPNAKGESMVSDGTVAIFIGIIMFIIPS...LLDWKTVNQKMPWN 420
NaDC3 ILFCMFAILLFTRDPKFIPGWASLFNPGFLSDA-VTGVAIVTILFFFPSQRPS...LLTWKKAQETVPWN 428
TM9b
H9c
HPout
HPout
VcINDY VLLLFGGGLCLSNVLKQTGTSVFLANALSDMVSHMGIFVVILVVATFVVFLTEFASNTASAALLIPVFAT 391
NaCT IVLLLGGGFALAKGSEASGLSVWMGKQMEPLHAVP-PAAITLILSLLVAVFTECTSNVATTTLFLPIFAS 478
NaDC1 IVLLLGGGYALAKGSERSGLSEWLGNKLTPLQSVPAPAIA-IILSLLVATFTECTSNVATTTIFLPILAS 489
NaDC3 IILLLGGGFAMAKGCEESGLSVWIGGQLHPLENVP-PALAVLLITVVIAFFTEFASNTATIIIFLPVLAE 497
* * * ***
TM10a
TM10b
TM11
VcINDY VAEAFGMSPVLLSVLIAVAASCAFMLPVATPPNAIVFASGHIKQSEMMRVGLYLNIACIGLLTAIAMLFW 461
NaCT MSRSIGLNPLYIMLPCTLSASFAFMLPVATPPNAIVFTYGHLKVADMVKTGVIMNIIGVFCVFLAVNTWG 548
NaDC1 MAQAICLHPLYVMLPCTLATSLAFMLPVATPPNAIVFSFGDLKVLDMARAGFLLNIIGVLIIALAINSWG 559
NaDC3 LAIRLRVHPLYLMIPGTVGCSFAFMLPVSTPPNSIAFASGHLLVKDMVRTGLLMNLMGVLLLSLAMNTWA 567

Supplementary Figure 1. Amino-acid sequence alignment of relevant DASS proteins.

Residues conserved among VcINDY and three human orthologues are colored magenta, regions of secondary structural elements in VcINDY are outlined. Red and blue dots highlight amino acids that bind succinate/citrate and Na^+, respectively. Positions for humanizing mutations carried by MT5, which include S200T, P201G, V322I, T379V, A376T, S381T, A382T and A383T, are marked by asterisks. For clarity, some residues in the human DASS proteins were omitted and indicated by "…". Notably, the amino-acid sequence identity between VcINDY and NaCT is 23%, but the degree of sequence conservation in and around the citrate- and Na^+-binding sites is substantially higher, suggesting that the VcINDY structure provides a useful model for studying the mechanism of NaCT.

a

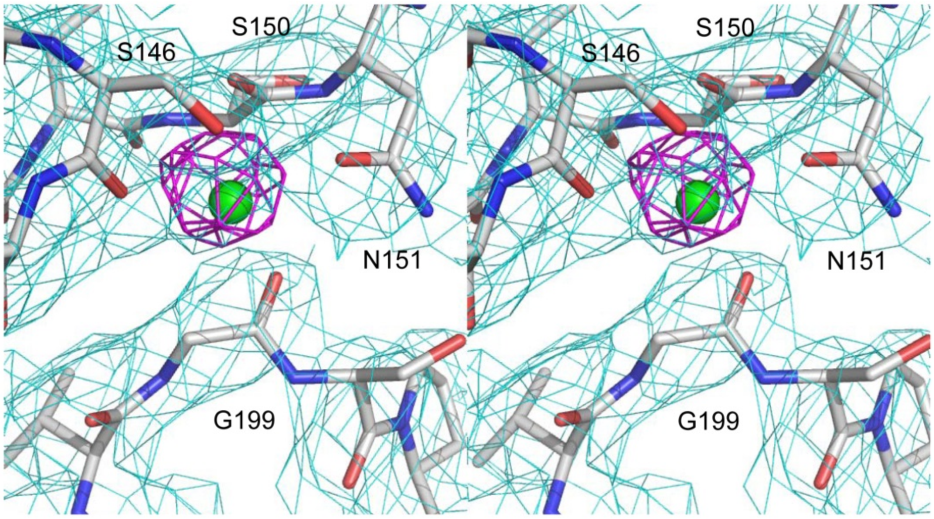

b

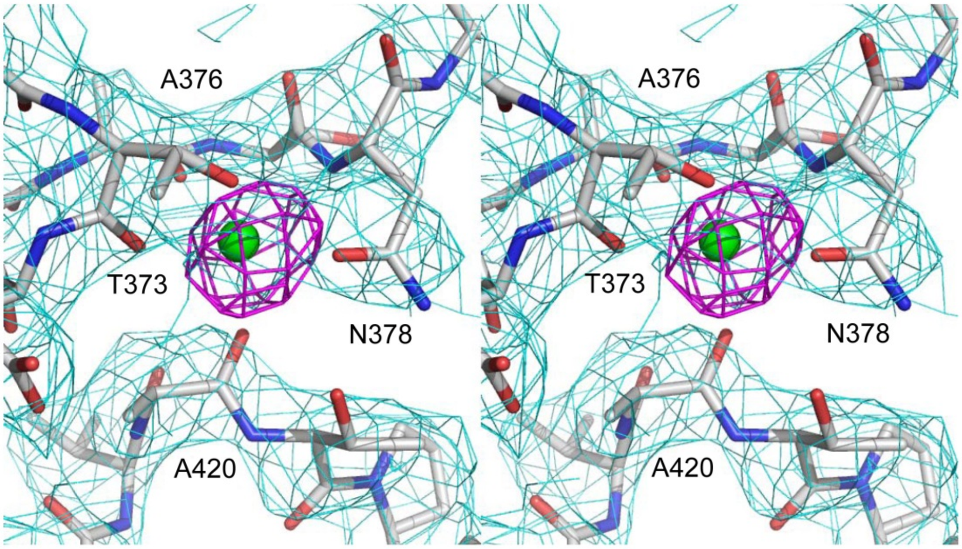

Supplementary Figure 2. Structure of the Na^+-binding sites in VcINDY.

Stereo view of the fitting of structural model to the electron density for the Na^+-binding sites Na1 (**a**) and Na2 (**b**). The experimental electron density map (cyan mesh, 1.2 σ) was calculated to 2.8 Å resolution using solvent-flattened MIRAS phases and overlaid onto the final model, whereas the F_o-F_c omit map was calculated to 2.8 Å resolution (magenta wire, 5 σ) using a VcINDY model that had never been refined with the bound Na^+ ion. Relevant amino acids and Na^+ ions are drawn as sticks models and green spheres, respectively.

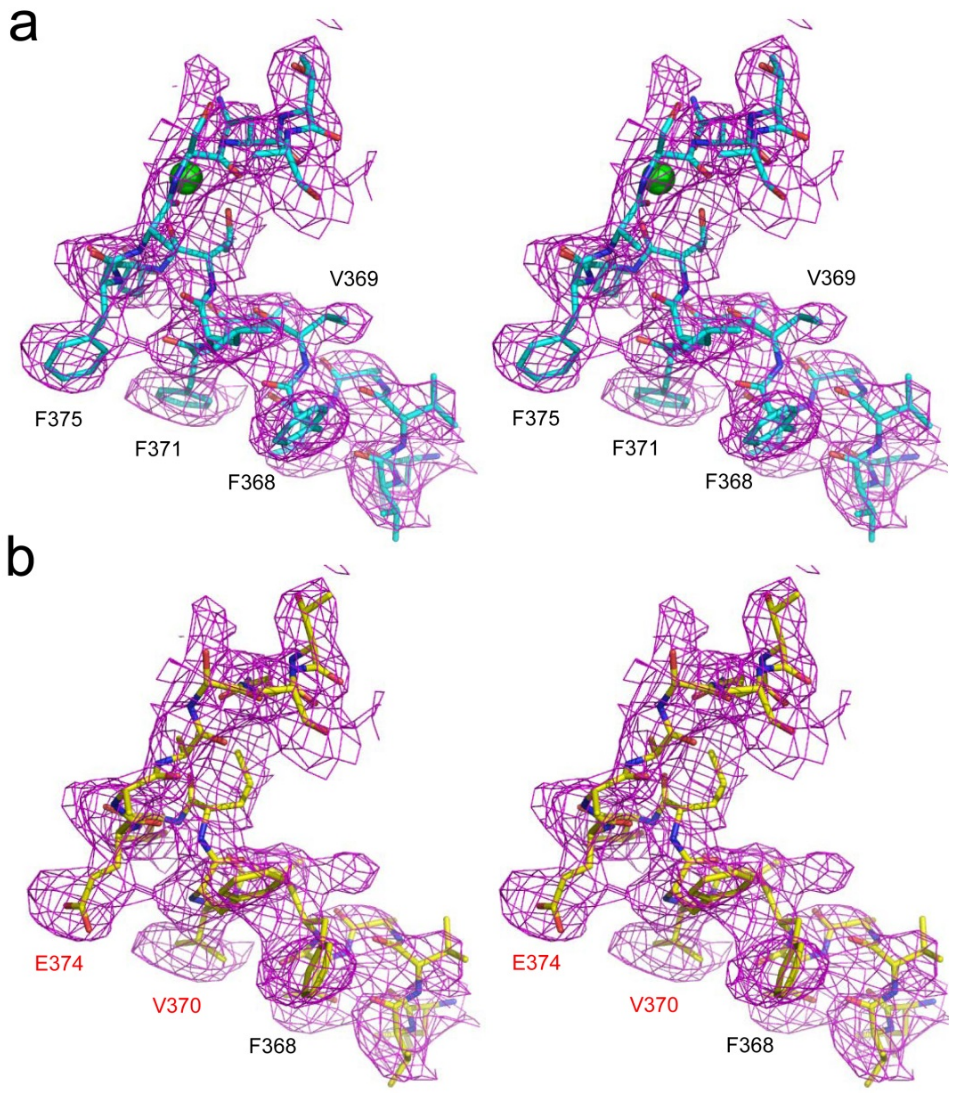

Supplementary Figure 3. Stereo views of the VcINDY models.

The experimental electron density map (magenta mesh, 1.2 σ) was calculated to 2.8 Å resolution using solvent-flattened MIRAS phases and overlaid onto the current model (**a**), or an earlier structure (**b**). Relevant amino acids and Na^+ are drawn as sticks models and a green sphere, respectively. This analysis suggested that residues 368-378 in PDB 4F35 were incorrectly modeled, which likely precluded the identification of Na2 in the previous work.

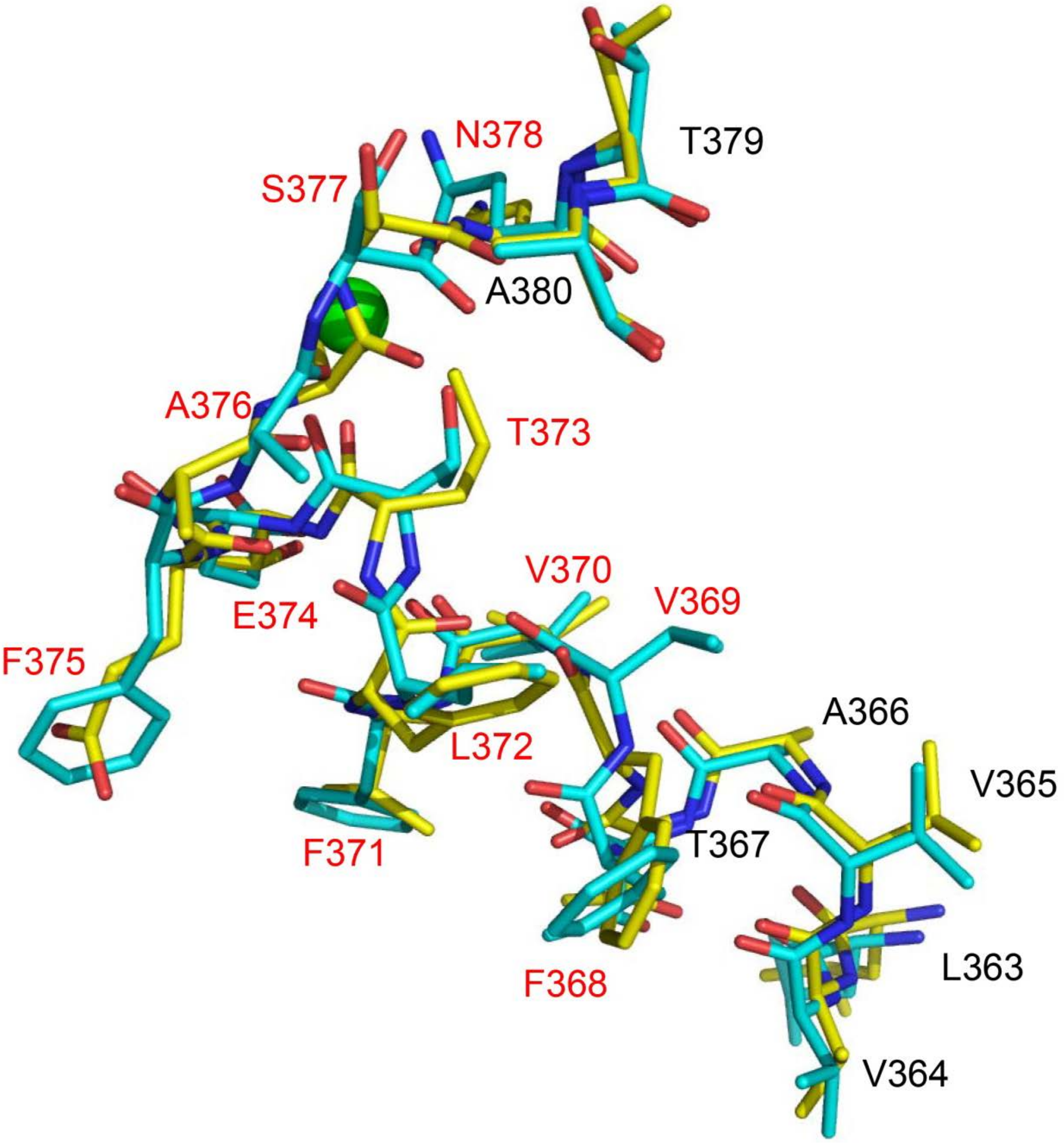

Supplementary Figure 4. Comparison of the structural models for VcINDY.

The current model for residues 363-379 is superimposed onto that of the previous work. Relevant amino acids and Na^+ (in Na2) are drawn as sticks models and a green sphere, respectively. The current model is colored cyan whereas the previous one is shown in yellow. Residues 368-378 in the current model are labeled in red to highlight the difference. The structural models are oriented similarly to those in Supplementary Figure 3.

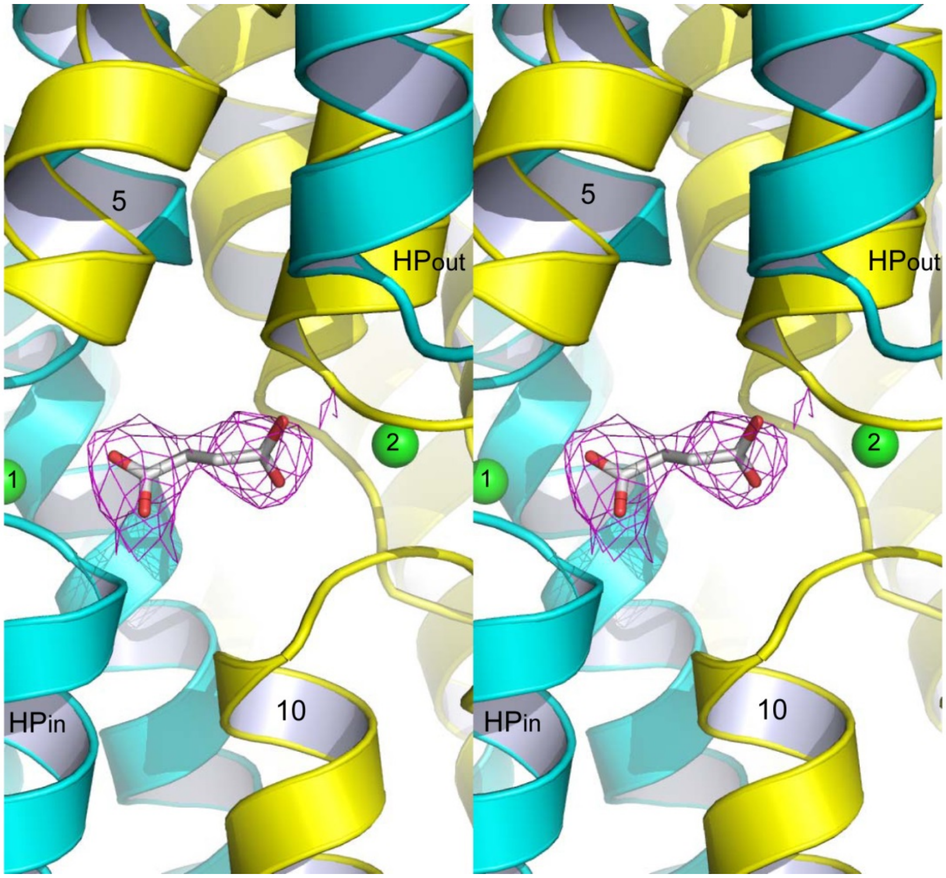

Supplementary Figure 5. Stereo view of the bound succinate in VcINDY.

The experimental electron density map (magenta mesh, 1.5 σ) was calculated to 2.8 Å resolution using solvent-flattened MIRAS phases and overlaid onto the final model of succinate (grey sticks). VcINDY is shown in ribbon rendition and two Na^+ ions are drawn as green spheres, respectively. Importantly, the quality of the experimental electron density map was sufficient for accurate placement of succinate.

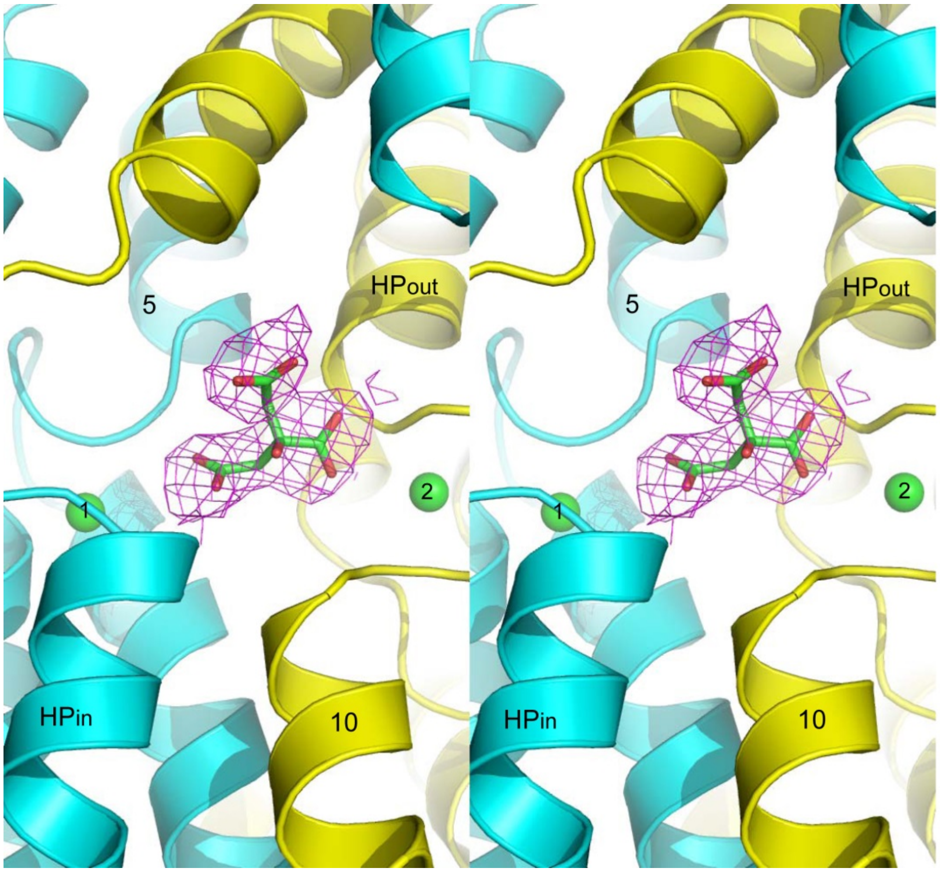

Supplementary Figure 6. Stereo view of the bound citrate in VcINDY.

The experimental electron density map (magenta mesh, 1.2 σ) was calculated to 2.8 Å resolution using solvent-flattened MIRAS phases and overlaid onto the final model of citrate (green sticks). VcINDY is displayed in ribbon diagram and two Na^+ ions are shown as green spheres, respectively. Notably, the high quality of the experimental electron density map allowed the placement of citrate with confidence.

a

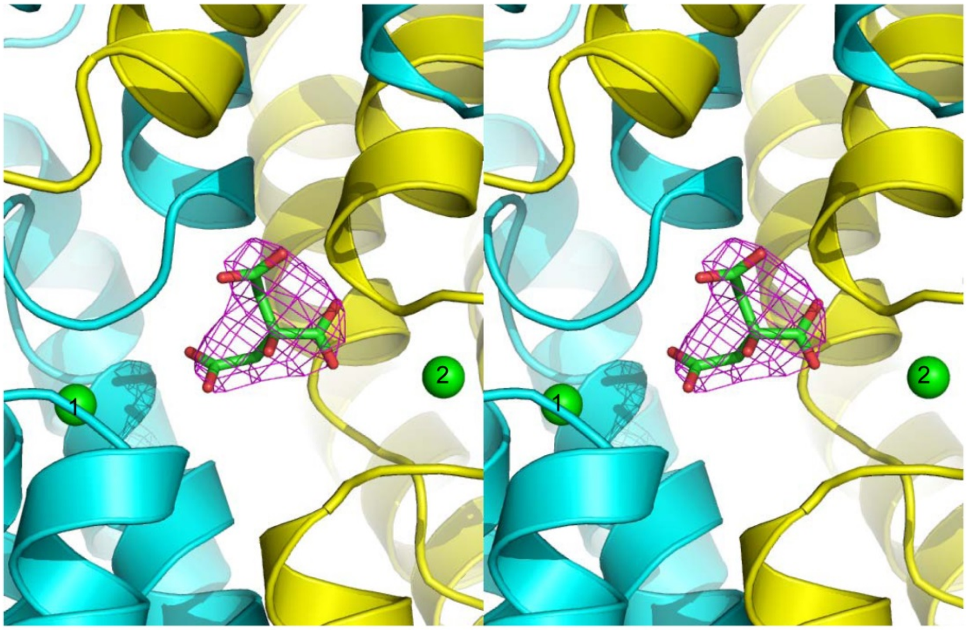

b

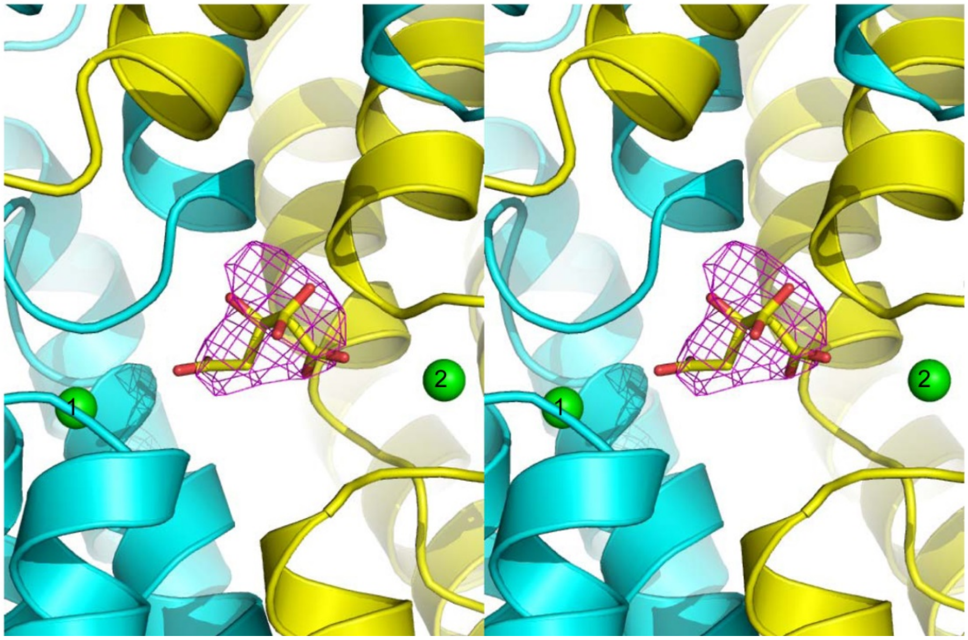

Supplementary Figure 7. Stereo views of the citrate models.

The F_o-F_c omit map (magenta mesh, 4 σ) was calculated using the published data (PDB 4F35) to 3.2 Å resolution and overlaid onto the citrate model either from the current structure (**a**), or from the previous structure (**b**). VcINDY is displayed in ribbon diagram and two Na^+ ions are drawn as green spheres, respectively. The VcINDY model used for the map calculation had never been refined with citrate in order to minimize model bias. This structural comparison highlighted that the citrate in PDB 4F35 was incorrectly modeled.

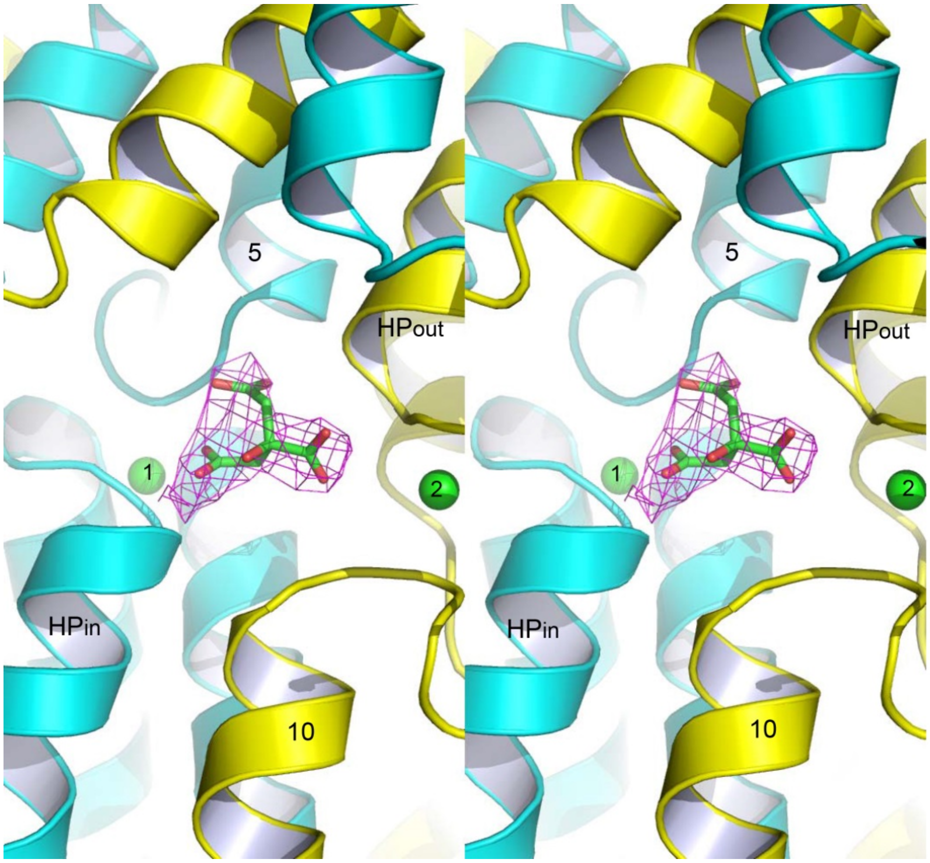

Supplementary Figure 8. Stereo view of the bound citrate in MT5.

The experimental electron density map (magenta mesh, 2.0 σ) was calculated to 2.8 Å resolution using solvent-flattened MIRAS phases and overlaid onto the final model of citrate (green sticks). Protein is displayed in ribbon diagram and two Na^+ ions are shown as green spheres, respectively. Of note, the high quality of the experimental electron density map enabled the accurate placement of citrate into the mutant, which is markedly different from that seen in VcINDY.

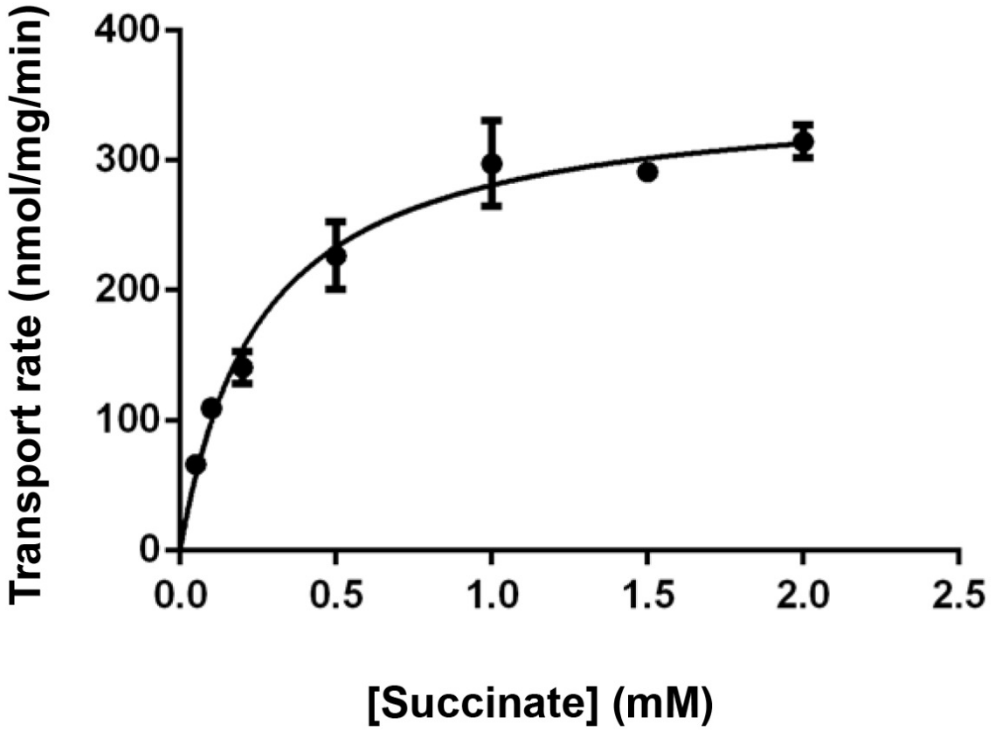

Supplementary Figure 9. Succinate transport activity of MT5.

Initial rates of succinate transport at 200 mM external Na^+ and pH 7.4 were plotted against the external succinate concentrations. Data were averaged to fit to the Michaelis-Menten equation, yielding a K_M of 0.26 mM and a V_{max} of 355 nmol mg^{-1} min^{-1}, respectively. Error bars represent s.d.. Notably, the K_M of MT5 is almost identical to that of VcINDY, whereas the V_{max} of MT5 is ~40% larger than that of VcINDY. This slight increase in V_{max} may be attributed to the experimental errors and/or the observation that MT5 makes fewer interactions with succinate than VcINDY, the latter of which implies that succinate may dissociate more readily from the inward-facing MT5 than VcINDY and thus gives rise to a higher transport rate. Error bars indicate standard deviations from at least 3 independent experiments.

Supplementary Table 1. Data collection and phasing statistics for succinate-bound VcINDY.

	Native	EMTS[a]	$K_2Pt(NO_2)_4$	K_2PtCl_4	$K_2Pt(NO_2)_4$ + EMTS
Wavelength	1.033 Å	1.008 Å	1.069 Å	1.069 Å	1.069 Å
Space Group	$P2_1$	$P2_1$	$P2_1$	$P2_1$	$P2_1$
a,b,c (Å)	106.68, 101.91, 167.74	106.43, 101.99, 166.95	105.22, 101.84, 165.59	106.38, 102.09, 166.28	105.08, 102.85, 166.99
Resolution	100-2.80 Å	100-3.10 Å	100-3.20 Å	100-3.40 Å	100-3.30 Å
Observations	1067123	1357219	1232535	694883	388792
Unique reflections	82858	64179	57620	48918	50425
Completeness (last shell)	99.9% (99.8%)	98.9% (85.4%)	99.8% (96.8%)	99.8% (97.9%)	96.7% (67.2%)
R_{sym}[b]	10.2%	11.7%	11.3%	10.7%	11.4%
(last shell)	(51.1%)	(55.9%)	(48.6%)	(53.2%)	(57.8%)
I/σ	21.0	22.6	24.9	21.0	18.0
(last shell)	(2.0)	(1.6)	(1.8)	(2.1)	(1.9)
Phasing power[c] (iso/ano)	N.A.	1.09/0.76	1.18/0.77	1.07/0.66	1.02/0.72
R_{cullis}[d] (iso/ano)	N.A.	0.69/0.86	0.61/0.82	0.67/0.92	0.74/0.88
Overall MIRAS figure of merit[e] (20-3.10 Å): 0.57 (acentric), 0.56 (centric).					

[a]EMTS: thimerosal.

[b]$R_{sym} = \Sigma| I - \langle I \rangle| / \Sigma I$, where I is the observed intensity of symmetry-related reflections.

[c]Phasing power= F_h / E, where F_h is the rms isomorphous/anomalous difference and E the rms residual lack-of-closure.

[d]R_{cullis}(iso)= $\Sigma(||FPH - FP| - |FH(calc)||) / \Sigma(|FPH - FP|)$, where FPH and FP are structure factors for derivative and native data, respectively. R_{cullis}(iso) is valid for centric reflections only.

[d]R_{cullis}(ano)= $\Sigma(||\Delta FPH(obs)| - |\Delta FPH(calc)||) / \Sigma|\Delta FPH(obs)|$, where ΔFPH(obs) and ΔFPH(calc) are the observed and calculated structure factor differences between Bijvoet pairs, respectively.

[e]Figure of merit is defined as weighted mean value of the cosine of phase error.

Supplementary Table 2. Data collection and phasing statistics for citrate-bound VcINDY.

	Native	EMTS[a]	$K_2Pt(NO_2)_4$	K_2PtCl_4	$K_2Pt(NO_2)_4$ + EMTS
Wavelength	1.000 Å	1.000 Å	1.000 Å	1.000 Å	1.000 Å
Space Group	$P2_1$	$P2_1$	$P2_1$	$P2_1$	$P2_1$
a,b,c (Å)	106.13, 102.11, 167.99	106.55, 102.27, 169.07	105.38, 102.47, 167.46	105.19, 101.84, 167.62	105.28, 102.57, 166.94
Resolution	100-2.80 Å	100-3.50 Å	100-3.00 Å	100-3.50 Å	100-3.50 Å
Observations	1248542	283548	367824	446051	339764
Unique reflections	82226	43489	58342	44526	43493
Completeness (last shell)	92.5% (58.1%)	97.3% (82.5%)	81.7% (30.8%)	96.0% (68.6%)	98.1% (89.2%)
R_{sym}[b]	10.3%	11.5%	8.8%	10.7%	10.2%
(last shell)	(48.8%)	(47.5%)	(38.0%)	(51.1%)	(46.2%)
I/σ	19.7	24.3	18.0	24.5	21.3
(last shell)	(1.8)	(1.8)	(1.5)	(2.1)	(1.7)
Phasing power[c] (iso/ano)	N.A.	1.21/0.81	1.08/0.67	1.01/0.76	1.04/0.73
R_{cullis}[d] (iso/ano)	N.A.	0.57/0.82	0.68/0.92	0.63/0.87	0.66/0.89
Overall MIRAS figure of merit[e] (20-3.00 Å): 0.51 (acentric), 0.49 (centric).					

[a]EMTS: thimerosal.

[b]$R_{sym} = \Sigma| I - \langle I\rangle| / \Sigma I$, where I is the observed intensity of symmetry-related reflections.

[c]Phasing power= F_h / E, where F_h is the rms isomorphous/anomalous difference and E the rms residual lack-of-closure.

[d]R_{cullis}(iso)= $\Sigma(||FPH – FP| - |FH(calc)||) / \Sigma(|FPH – FP|)$, where FPH and FP are structure factors for derivative and native data, respectively. R_{cullis}(iso) is valid for centric reflections only.

[d]R_{cullis}(ano)= $\Sigma(||\Delta FPH(obs)| - |\Delta FPH(calc)||) / \Sigma|\Delta FPH(obs)|$, where ΔFPH(obs) and ΔFPH(calc) are the observed and calculated structure factor differences between Bijvoet pairs, respectively.

[e]Figure of merit is defined as weighted mean value of the cosine of phase error.

Supplementary Table 3. Data collection and phasing statistics for citrate-bound MT5.

	Native	EMTS[a]	$K_2Pt(NO_2)_4$	K_2PtCl_4	$K_2Pt(NO_2)_4$ + EMTS
Wavelength	1.000 Å	1.000 Å	1.000 Å	1.000 Å	1.000 Å
Space Group	$P2_1$	$P2_1$	$P2_1$	$P2_1$	$P2_1$
a,b,c (Å)	106.09, 101.54, 168.89	105.23, 101.33, 168.17	105.87, 101.55, 167.01	106.07, 101.47, 166.96	104.69, 101.84, 166.88
Resolution	100-2.80 Å	100-3.00 Å	100-3.20 Å	100-3.40 Å	100-3.20 Å
Observations	2382353	1808948	1232348	423304	1027370
Unique reflections	87891	66983	57569	47380	57968
Completeness (last shell)	99.5% (92.5%)	95.0% (59.1%)	99.9% (98.4%)	96.8% (85.0%)	99.1% (84.1%)
R_{sym}[b]	11.1%	13.8%	13.4%	12.2%	14.0%
(last shell)	(53.6%)	(61.0%)	(71.6%)	(65.3%)	(62.0%)
I/σ	27.6	18.2	23.9	19.9	24.2
(last shell)	(2.1)	(1.4)	(1.6)	(1.2)	(1.8)
Phasing power[c] (iso/ano)	N.A.	1.03/0.71	1.11/0.77	1.00/0.66	1.01/0.70
R_{cullis}[d] (iso/ano)	N.A.	0.67/0.87	0.63/0.84	0.73/0.97	0.69/0.83

Overall MIRAS figure of merit[e] (20-3.00 Å): 0.54 (acentric), 0.51 (centric).

[a]EMTS: thimerosal.

[b]$R_{sym} = \Sigma| I - \langle I \rangle | / \Sigma I$, where I is the observed intensity of symmetry-related reflections.

[c]Phasing power= F_h / E, where F_h is the rms isomorphous/anomalous difference and E the rms residual lack-of-closure.

[d]R_{cullis}(iso)= Σ(||FPH – FP| - |FH(calc)||) / Σ(|FPH – FP|), where FPH and FP are structure factors for derivative and native data, respectively. R_{cullis}(iso) is valid for centric reflections only.

[d]R_{cullis}(ano)= Σ(||ΔFPH(obs)| - |ΔFPH(calc)||) / Σ|ΔFPH(obs)|, where ΔFPH(obs) and ΔFPH(calc) are the observed and calculated structure factor differences between Bijvoet pairs, respectively.

[e]Figure of merit is defined as weighted mean value of the cosine of phase error.

Supplementary Table 4. Data collection and phasing statistics for succinate-bound MT5.

	Native	EMTS[a]	$K_2Pt(NO_2)_4$	K_2PtCl_4	$K_2Pt(NO_2)_4$ + EMTS
Wavelength	1.000 Å	1.000 Å	1.000 Å	1.000 Å	1.000 Å
Space Group	$P2_1$	$P2_1$	$P2_1$	$P2_1$	$P2_1$
a,b,c (Å)	107.14, 102.28, 170.86	105.22, 101.98, 168.55	105.16, 102.09, 169.01	107.48, 102.30, 169.38	104.39, 103.56, 167.45
Resolution	100-2.80 Å	100-3.20 Å	100-3.40 Å	100-3.50 Å	100-3.50 Å
Observations	2110364	690534	935056	886364	394908
Unique reflections	86060	58462	48650	46191	43921
Completeness (last shell)	96.4% (61.8%)	99.1% (89.7%)	97.9% (68.5%)	99.5% (92.2%)	98.7% (92.3%)
R_{sym}[b]	10.5%	11.9%	13.7%	13.0%	10.7%
(last shell)	(59.6%)	(50.3%)	(65.8%)	(52.3%)	(50.2%)
I/σ	26.2	27.5	23.7	40.6	23.5
(last shell)	(1.9)	(1.7)	(1.8)	(1.3)	(1.4)
Phasing power[c] (iso/ano)	N.A.	1.13/0.76	1.11/0.71	1.21/0.96	1.07/0.70
R_{cullis}[d] (iso/ano)	N.A.	0.63/0.87	0.65/0.91	0.54/0.67	0.70/0.92
Overall MIRAS figure of merit[e] (20-3.20 Å): 0.57 (acentric), 0.53 (centric).					

[a]EMTS: thimerosal.

[b]$R_{sym} = \Sigma| I - \langle I \rangle | / \Sigma I$, where I is the observed intensity of symmetry-related reflections.

[c]Phasing power= F_h / E, where F_h is the rms isomorphous/anomalous difference and E the rms residual lack-of-closure.

[d]$R_{cullis}(iso)= \Sigma(||FPH – FP| - |FH(calc)||) / \Sigma(|FPH – FP|)$, where FPH and FP are structure factors for derivative and native data, respectively. R_{cullis}(iso) is valid for centric reflections only.

[d]$R_{cullis}(ano)= \Sigma(||\Delta FPH(obs)| - |\Delta FPH(calc)||) / \Sigma|\Delta FPH(obs)|$, where ΔFPH(obs) and ΔFPH(calc) are the observed and calculated structure factor differences between Bijvoet pairs, respectively.

[e]Figure of merit is defined as weighted mean value of the cosine of phase error.
